# Supplementary material for: Direct Patterning and Spontaneous Self-Assembly of Graphene Oxide via Electrohydrodynamic Jet Printing for Energy Storage and Sensing
Source: Micromachines (Basel). 2019 Dec 19;11(1):13. doi: 10.3390/mi11010013 (PMC7019724; doi:10.3390/mi11010013)
Supplement: Supplementary file 1 [file micromachines-11-00013-s001.pdf]

micromachines

Supplementary Materials: Direct Patterning and Spontaneous Self-Assembly of Graphene Oxide via Electrohydrodynamic Jet Printing for Energy Storage and Sensing

Bin Zhang, Jaehyun Lee, Mincheol Kim, Naeeung Lee, Hyungdong Lee, and Doyoung Byun

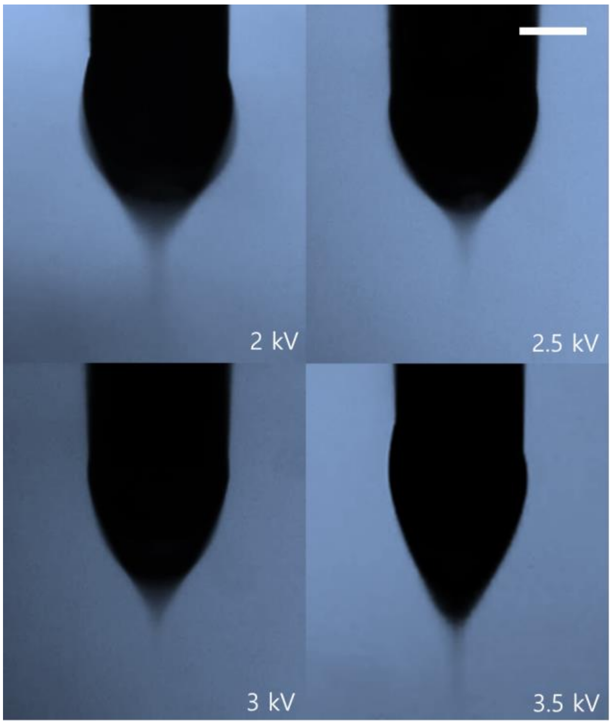

Figure S1. The effect of voltage on the thickness of the jet fiber. The scale bar is 100 μm.

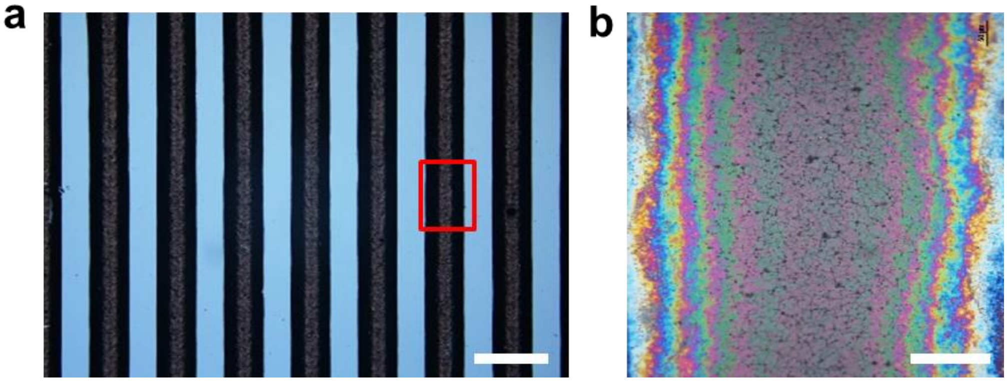

Figure S2. (**a**) EHD jet printed linear pattern. The scale bar is 500 μm. (**b**) The magnification image in (a) shows the coffee ring effect. The color of the pattern is gradually changed by different of the GO thickness. The scale bar is 100 μm.

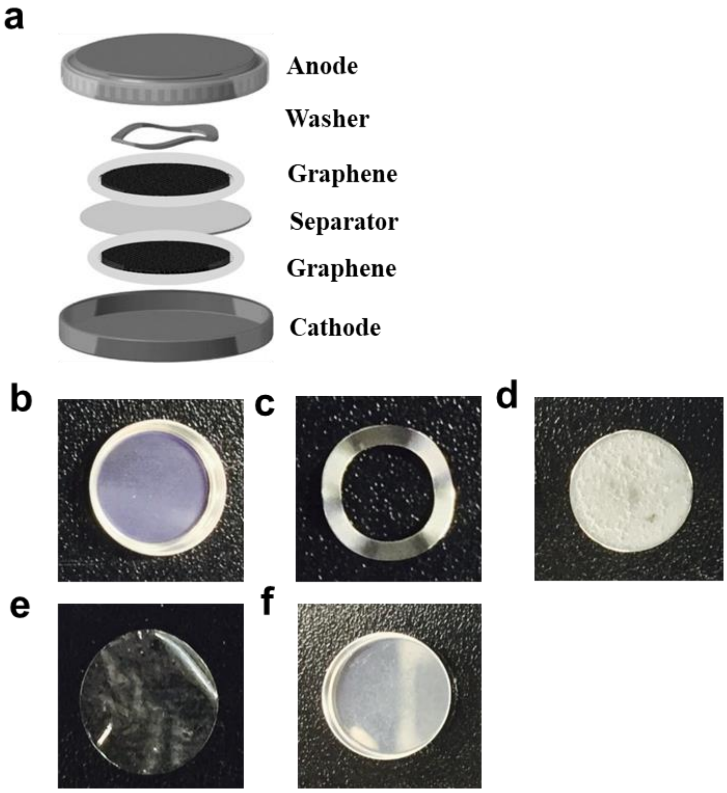

Figure S3. Structure of coin cell type supercapacitor. (**a**) schematic illustration of a coin cell type supercapacitor. (**b**) A photo of the anode. (**c**) Wave washer. (**d**) Graphene printed on the electrode. (**e**) Polymer electrolyte (PVA/H_3PO_4). (**f**) Cathode.

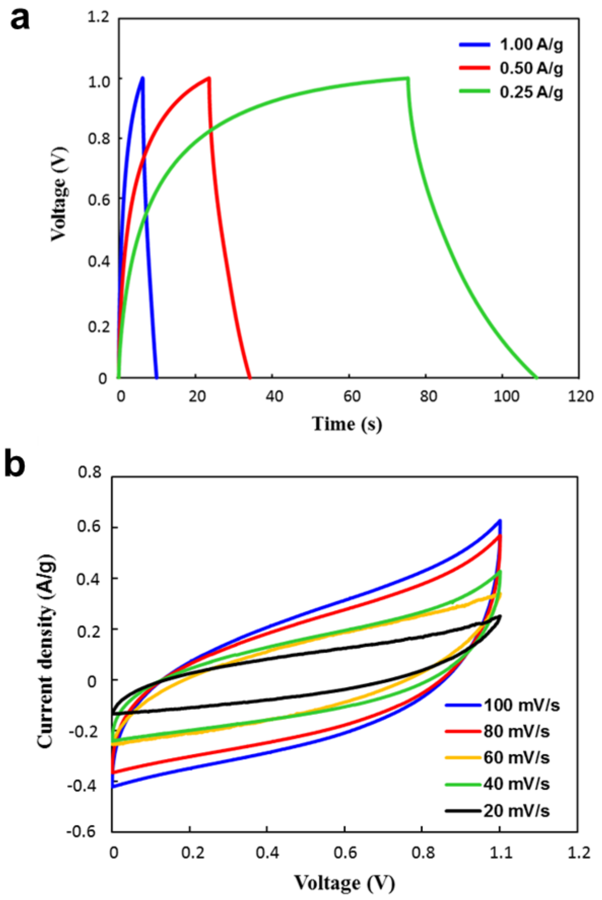

Figure S4. Properties of a two-electrode coin cell type supercapacitor. (**a**) Galvanostatic charge/discharge testing results at a current density from 0.25 to 1 A/g. (**b**) Cyclic voltammetry (CV) diagram with varying scan rate from 20 to 100 mV/s.

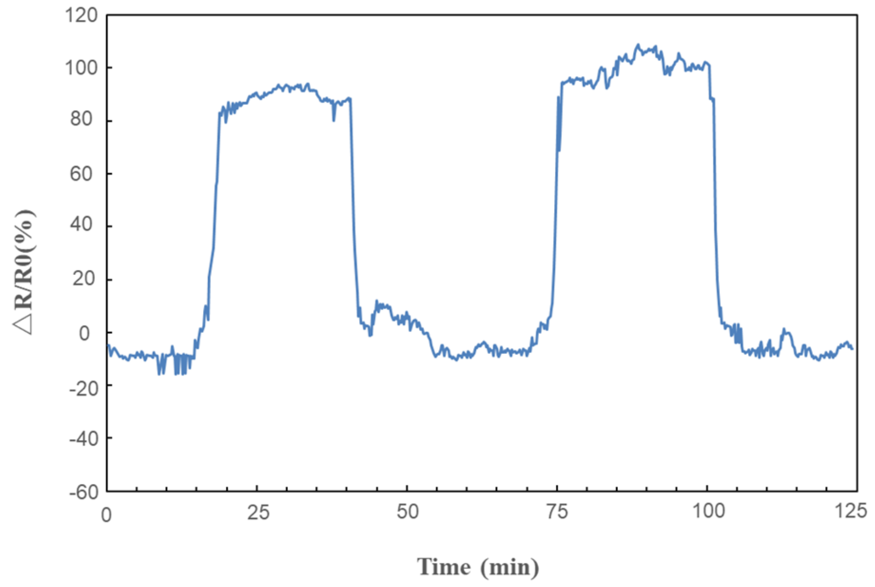

Figure S5. The saturation curve of the high aspect ratio sensor.
